# Pharmacological Characterization of Veldoreotide as a Somatostatin Receptor 4 Agonist

**DOI:** 10.3390/life11101075

**Published:** 2021-10-12

**Authors:** Pooja Dasgupta, Thomas Gűnther, Stefan Schulz

**Affiliations:** Institute of Pharmacology and Toxicology, Jena University Hospital, Friedrich Schiller University Jena, 07747 Jena, Germany; Pooja.Dasgupta@med.uni-jena.de (P.D.); qm-thomas-guenther@web.de (T.G.)

**Keywords:** somatostatin receptor 4, agonist, pain, receptor signaling, somatostatin analogue

## Abstract

Veldoreotide, a somatostatin analogue, binds to the somatostatin receptors (SSTR) 2, 4, and 5. The current aim was to assess its pharmacological activity as an SSTR4 agonist. G-protein signaling was assessed using a fluorescence-based membrane potential assay in human embryonic kidney 293 (HEK293) cells stably co-expressing G-protein-coupled inwardly rectifying potassium 2 channels and the individual SSTR2, SSTR4, and SSTR5, and in human BON-1 cells stably expressing these SSTRs. Veldoreotide effects on chromogranin A (CgA) secretion and cell proliferation were examined in BON-1 cells. In HEK293 transfected cells, veldoreotide showed a high efficacy for activating the SSTR4; octreotide and pasireotide had little activity (E_max_, 99.5% vs. 27.4% and 52.0%, respectively). Veldoreotide also activated SSTR2 and SSTR5 (E_max_, 98.4% and 96.9%, respectively). In BON-1 cells, veldoreotide activated SSTR2, SSTR4, and SSTR5 with high potency and efficacy. CgA secretion was decreased to a greater degree in the BON-1 cells expressing SSTR4 versus the cells expressing SSTR2 and SSTR5 (65.3% vs. 80.3% and 77.6%, respectively). In the BON-1 cells expressing SSTR4, veldoreotide inhibited cell proliferation more than somatostatin SS-14 (71.2% vs. 79.7%) and to a similar extent as the SSTR4 agonist J-2156 in the presence of SSTR2 and SSTR5 antagonists. Veldoreotide is a full agonist of SSTR2, SSTR4, and SSTR5.

## 1. Introduction

The neuropeptide somatostatin (SST) inhibits the secretion of numerous hormones in the human body (e.g., growth hormone, glucagon, and insulin) [1] through its interactions with the 5 G-protein-coupled SST receptors (SST receptors 1–5) [2]. Somatostatin also inhibits tumor growth by its antiproliferative activity and by inducing apoptosis [3]. Stable SST analogs (SSAs) have been developed for clinical use given that naturally occurring SST has a very short half-life (<3 min) [2]. The SSAs octreotide, lanreotide, and pasireotide have been approved for treatment of patients in endocrinology (e.g., acromegaly, Cushing’s disease, and neuroendocrine tumors) and gastroenterology settings (e.g., prevention of postoperative complications of pancreatic surgery and upper gastrointestinal bleeding related to gastrointestinal varices in cirrhosis) [1].

The binding affinity for SST receptors differentiates the SSAs. Octreotide and lanreotide bind to the SST2 receptor with high affinity, to the SST3 and SST5 receptors with moderate affinity, but have little-to-no affinity for the SST1 and SST4 receptors [4]. Pasireotide binds to all SST receptors except SST4 with high affinity [5]. Veldoreotide, a novel SSA currently under evaluation in preclinical and clinical studies, binds to the SST2, SST4, and SST5 receptors [6,7,8]. However, very little is known about the SST4-mediated in vitro effects of veldoreotide.

The role of the SST4 receptor in the human body is not entirely clear, but its expression has been shown in the brain, the duodenum, the kidney, the lung, the non-islet cells of the pancreas, the parathyroid gland, the placenta, the stomach, and the T cells [9,10,11,12,13,14,15]. The protective role of this receptor was demonstrated in experimental models of chronic arthritis, airway inflammation, and skin hypersensitivity in mice lacking the SST4 receptor, with knock-out mice presenting with more-severe contact dermatitis, increased lung edema and bronchial inflammation, and greater inflammatory hyperalgesia compared with their SST4 receptor-expressing littermates [16]. Patients with chronic pulmonary inflammation were shown to have an increased expression of SST4 receptors [11]. Thus, several lines of evidence suggest that SST mediates anti-inflammatory and antihyperalgesic effects through the SST4 receptor [16,17,18,19]. Expression of the SST4 receptor has also been shown in pituitary tumors producing growth hormone, malignant pleural mesothelioma, and breast and thyroid tumors [20,21,22,23].

Given that most SSAs have low-to-no binding affinity for the SST4 receptor, it is noteworthy that veldoreotide binds to this receptor [6,7]. In this study, the in vitro pharmacological activity of veldoreotide at the SST4 receptor was characterized by assessing G-protein signaling via a novel, fluorescent-based membrane potential assay and antisecretory and antiproliferative activities of veldoreotide.

## 2. Materials and Methods

### 2.1. DNA Constructs

Plasmid pcDNA 3.1 encoding the SST2, SST4, and SST5 receptors was obtained from cDNA.org. Amino terminal hemagglutinin (HA) tags or T7 tags were added to SST2, SST4, and SST5 receptor DNA for eventual selection and enrichment of stable transfected cells. A custom-made plasmid pCMV vector encoding the human G-protein-coupled inwardly rectifying potassium 2 (GIRK2) channel along with a green fluorescence protein (GFP) tag were obtained from OriGene (Rockville, MD, USA), as previously described [24].

### 2.2. Cell Culture and Transfection

Human embryonic kidney 293 (HEK293) cells were obtained from the DSMZ-German Collection of Microorganisms and Cell Cultures GmbH (Braunschweig, Germany), and human pancreatic neuroendocrine tumor BON-1 cells were kindly provided by Dr. Herbert Schmid (Novartis, Basel, Switzerland). HEK293 cells were cultured in Dulbecco’s Modified Eagle’s Medium (DMEM) and supplemented with 10% fetal bovine serum (FBS), 2 mM L-glutamine, and 100 U/mL penicillin/streptomycin at 37 °C and 5% CO_2_. BON-1 cells were cultured in DMEM and Ham’s F12 medium (1:1 ratio) supplemented with 10% FBS, 2 mM L-glutamine, and 100 U/mL penicillin/streptomycin at 37 °C and 5% CO_2_.

HEK293 and BON-1 cell lines were stably transfected with the pcDNA 3.1 vectors encoding human SST2 or SST5 receptors or a rat SST4 receptor using TurboFect Transfection Reagent (Thermo Fisher Scientific, Schwerte, Germany). Stably transfected cells were selected in a medium supplemented with 400 µg/mL geneticin. HEK293 cells were cotransfected with vector encoding GFP-tagged GIRK2 channels, and stably transfected cells were selected in the presence of 300 µg/mL hygromycin B. Fluorescence-activated cell sorting was used as previously described [24,25,26] to obtain a homogenous level of SST2, SST4, or SST5 receptor expression in HEK293 or BON-1 cells.

### 2.3. Drugs and Chemicals

**Figure 1:**
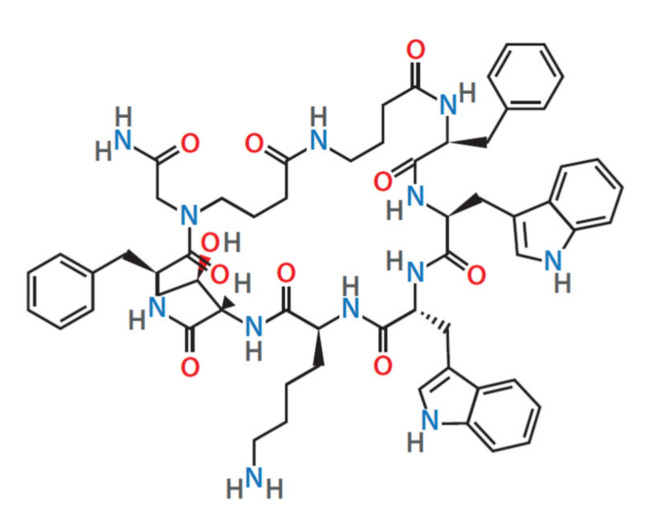
Chemical structure of veldoreotide.

Somatostatin SS-14 (4033009), octreotide, and pasireotide were obtained from Bachem (UK) Ltd. (St. Helens, UK). Veldoreotide (Figure 1) was obtained from Strongbridge Biopharma (Trevose, PA, USA). J-2156 was obtained from Juvantia Pharma Ltd. (Turku, Finland). SST2 and SST5 receptor antagonists BIM23454 and BIM23867, respectively, were obtained from Ipsen Bioscience (Cambridge, MA, USA).

### 2.4. Immunohistochemistry

Stably transfected HEK293 and BON-1 cells were seeded on poly-L-lysine-coated coverslips for 24 h and incubated with either rabbit anti-HA antibody (0631) or rabbit anti-T7 antibody (Gramsch Laboratories, Schwabhausen, Germany) in Opti-MEM medium (Thermo Fisher Scientific) at 4 °C for an hour [27]. Cells were fixed for 20 min at room temperature using 4% paraformaldehyde and 0.2% picric acid in a phosphate buffer (pH 6.9) and blocked with 3% normal goat serum in a phosphate buffer. Subsequently, cells were incubated with Alexa 488-conjugated anti-rabbit antibody (Life Technologies, Darmstadt, Germany) or Cy3-coupled anti-rabbit antibody (Life Technologies) and imaged using an LSM 510 META laser scanning confocal microscope (Carl Zeiss, Jena, Germany).

### 2.5. Membrane Potential Assay

G-protein signaling was measured using a membrane potential assay as previously described [24]. Briefly, cells were seeded in poly-L-lysine coated 96-well plates and grown at 37 °C and 5% CO_2_ for 48–72 h to 70–80% confluency. Cells were washed with Hank’s Balanced Salt Solution (HBSS; 1.3 mM CaCl_2_, 5.4 mM KCl, 0.4 K_2_HPO_4_, 0.5 mM MgCl_2_, 0.4 mM MgSO_4_, 136.9 mM NaCl, 0.3 mM Na_2_HPO_4_, 4.2 mM NaHCO_3_, and 5.5 mM glucose) buffered with 20 mM HEPES solution (pH 7.4), and 90 µL HBSS/HEPES buffer solution plus 90 µL reconstituted membrane potential dye (FLIPR Membrane Potential Assay Kit Blue, Molecular Devices, Biberach, Germany) were added to the cells, which were then incubated at 37 °C for 45 min.

Test compounds were prepared in HBSS buffered with 20 mM HEPES solution (pH 7.4) at 10 times the final concentration to be measured. Fluorescence measurements were performed using a FlexStation 3 microplate reader (Molecular Devices) at 37 °C, using excitation and emission wavelengths of 530 nm and 565 nm, respectively. Baseline readings were obtained every 1.8 s for 60 s. After 60 s, 20 µL 10× test compound or solvent was injected into each well for a final well volume of 200 µL. The change in dye fluorescence was recorded for 240 s using SoftMax Pro software (Molecular Devices). The data obtained were normalized to baseline and graphed using OriginPro 2015 (OriginLab Corporation, Northampton, MA, USA) to generate concentration-response curves.

### 2.6. Enzyme-Linked Immunosorbent Assay (ELISA)

Concentrations of human CgA secreted by BON-1 cells treated with 10 µM SST agonists for 3 h at 37 °C were determined using the Human Chromogranin A ELISA kit (ab196271, Abcam, Berlin, Germany) per the manufacturer’s instructions. Absorbance was measured at 450 nm using a FlexStation 3 microplate reader (Molecular Devices).

### 2.7. Cell Proliferation Assay

Cell proliferation was examined using the colorimetric WST-1 assay (Cayman Chemical, Ann Arbor, MI, USA). BON-1 cells were plated in normal growth medium in 96-well plates (10^4^ to 10^5^ cells/well) and grown at 37 °C and 5% CO_2_ for 48 h. To each well, 100 µL of Opti-MEM medium (Thermo Fisher Scientific) containing agonists (i.e., SS-14, veldoreotide, or J-2156) was added for a final concentration of 10 µM for 24 h. After 24 h, 10 µL WST-1 reagent was added to each well, and the plates were incubated at 37 °C for 2 h. Absorbance was measured at a wavelength of 450 nm using a FlexStation 3 microplate reader (Molecular Devices).

### 2.8. Statistical Analysis

All data are represented as mean ± standard error of the mean. Statistical significance was analyzed between the control and the test compounds using Dunnet’s multiple comparisons test following a one-way analysis of variance. The significance level was set at 0.05.

## 3. Results

### 3.1. SST Receptor and GIRK2 Channel Expression

**Figure 2:**
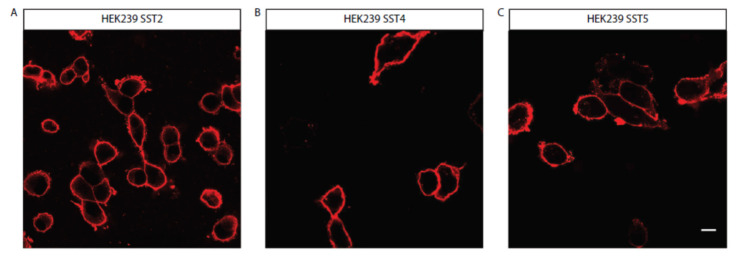
Stable expression of (**A**) SST2, (**B**) SST4, or (**C**) SST5 receptors in HEK293 cells. Red staining (Cy3) confirms SST receptor expression in cells. 40× magnification; bar = 20 μm. SST = somatostatin.

**Figure 3:**
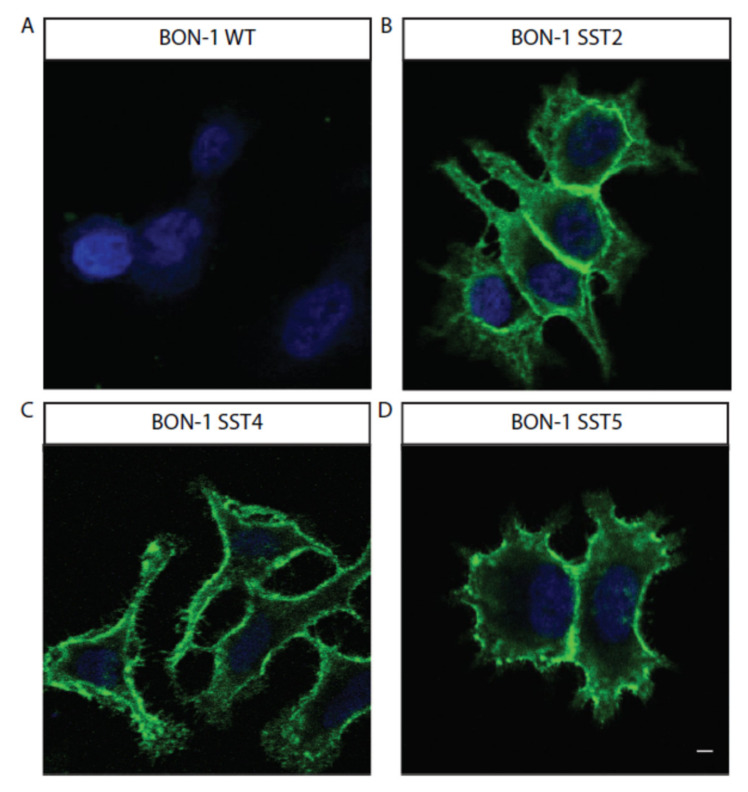
Stable expression of SST receptors in BON-1 cells: (**A**) wild-type cells (**B**) SST2 receptor, (**C**) SST4 receptor, or (**D**) SST5 receptor. Green staining (Alexa488) confirms SST receptor expression in cells. 63× magnification; bar = 20 μm. SST = somatostatin.

The human HEK293 cells were stably transfected with GFP-tagged GIRK2 channel-expressing plasmids and individual SST (SST2, SST4, and SST5)-receptor-expressing plasmids. The BON-1 cells were also stably transfected with these individual SST-receptor-encoding plasmids. The expression of SST receptors in the HEK293 and BON-1 cells (Figure 2 and Figure 3) and the cotransfected GFP-GIRK2 channels in the HEK293 cells (Figure S1) were confirmed using immunocytochemistry. The individual SST-receptor expression was mostly confined to the plasma membrane in both cell types.

### 3.2. Ligand-Induced Receptor Activation

**Figure 4:**
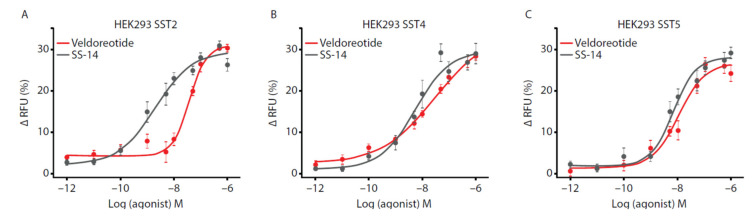
Analysis of exogenous (**A**) SST2, (**B**) SST4, or (**C**) SST5 receptors co-expressed with GIRK2 channels in HEK293 cells. Cells were exposed to 10^−12^ M to 10^−6^ M SS-14 or veldoreotide. Concentration-response curves were generated using data from three to four independent experiments performed in duplicate (mean ± SEM). Changes in fluorescence with agonists were subtracted from changes in fluorescence with vehicle. GIRK = G-protein-coupled inwardly rectifying potassium; RFU = relative fluorescence units; SEM = standard error of the mean; SST = somatostatin.

A fluorescence-based membrane potential assay [24] examined the activation of the co-expressed individual SST receptors and the GIRK2 channels in the HEK293 cells following treatment with SS-14, octreotide, pasireotide, and veldoreotide (representative traces shown in Figure S2). The concentration-response curves revealed that veldoreotide, similar to SS-14, stimulated the SST2, SST4, and SST5 receptors with high potency and efficacy in the HEK293 cells, co-expressing these receptors with the GIRK2 channels (Figure 4; Table 1). The half-maximal effective concentration (EC_50_) of veldoreotide was between that of octreotide and pasireotide for activating the SST2 receptor and was comparable with pasireotide for activating the SST5 receptor. Veldoreotide revealed a high efficacy for activating the SST4 receptor based on the effective maximum (E_max_) values, in contrast with octreotide and pasireotide. The E_max_ values of the SST4 receptor agonist J-2156 at the SST4 receptor in the HEK293 cells stably expressing this receptor were similar to those observed with veldoreotide.

**Figure 5:**
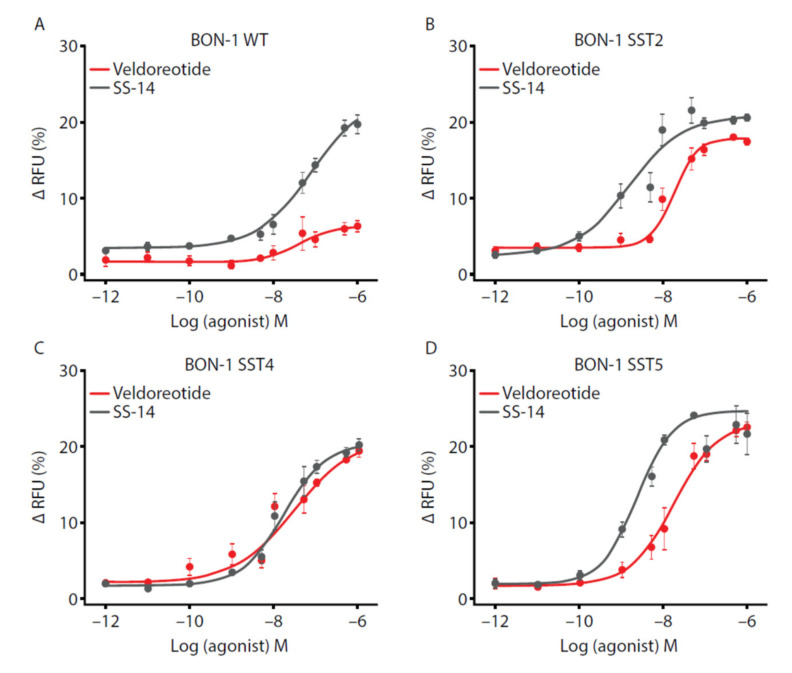
Analysis of exogenous (**A**) wild-type, (**B**) SST2, (**C**) SST4, or (**D**) SST5 receptors in BON-1 cells. Cells were exposed to 10^−12^ M to 10^−6^ M SS-14 or veldoreotide. Concentration-response curves were generated using data from four independent experiments performed in duplicate (mean ± SEM). Changes in fluorescence with agonists were subtracted from changes in fluorescence with vehicle. RFU = relative fluorescence units; SEM = standard error of the mean; SST = somatostatin.

G-protein signaling also was assessed using the fluorescence-based membrane potential assay in the BON-1 cells. In the BON-1 cells stably expressing the SST2 receptor, veldoreotide had a steeper concentration-response curve compared with SS-14 (Figure 5). The high potency and efficacy of veldoreotide for the SST4 and SST5 receptors were shown in the BON-1 cells stably expressing these receptors; these findings are in contrast with the wild-type BON-1 cells, which express SST2 and SST5 receptors endogenously. The wild-type BON-1 cells showed low levels of fluorescence change over a broad range of dosing (10^−12^ M to 10^−8^ M), with a slight increase from 10^−8^ M to 10^−6^ M veldoreotide concentration, that did not achieve the fluorescence levels observed with the BON-1 cells expressing the SST2, SST4, or SST5 receptors. The efficacy of veldoreotide for SST4 receptor activation was comparable with that of SS-14, as determined by E_max_ values (Table 2). Furthermore, the E_max_ of veldoreotide was greater for the SST4 receptor (96.5%) as compared to the SST2 receptor (83.1%); however, activation of the SST5 receptor was comparable with that of the SST4 receptor (93.3%; Table 2). The EC_50_ of veldoreotide was lowest for the SST2 and SST5 receptors; the EC_50_ for activating the SST4 receptor was between that of the SST2 and SST5 receptors and the wild-type endogenous receptors (Table 2). The E_max_ values of J-2156 at the SST4 receptor in the BON-1 cells stably expressing this receptor were similar to those observed with veldoreotide.

### 3.3. Chromogranin A Secretion

**Figure 6:**
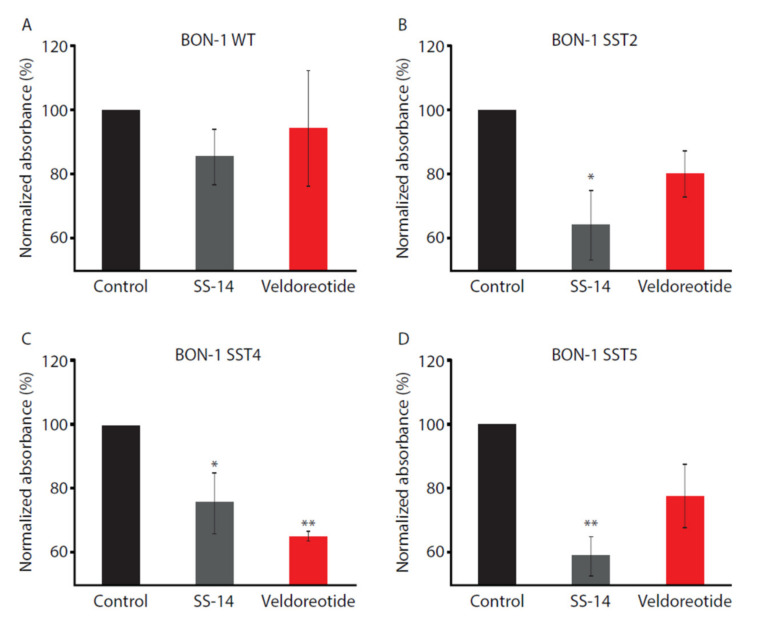
Chromogranin A secretion in (**A**) wild-type BON-1 cells or in BON-1 cells expressing (**B**) SST2, (**C**) SST4, or (**D**) SST5 receptors treated with 10 µM SS-14 or 10 µM veldoreotide for 3 h at 37 °C. Data shown were generated from four independent experiments performed in duplicate (mean ± SEM). Absorbance data for cells treated with agonists were normalized to vehicle (control). * *p* < 0.05 vs. control; ** *p* < 0.01 vs. control. SEM = standard error of the mean.

Chromogranin A, a secretory protein, is a well-established biomarker for neuroendocrine tumors [28]. Veldoreotide had no effect on the CgA secretion in the wild-type BON-1 cells (Figure 6) but inhibited the CgA release in the BON-1 cells stably expressing the SST2, SST4, and SST5 receptors. Notably, the BON-1 cells stably expressing the SST4 receptor had the greatest reduction in CgA secretion with veldoreotide when compared with the BON-1 cells stably expressing the SST2 and SST5 receptors (65.3% vs. 80.3% and 77.6%, respectively).

### 3.4. Cell Proliferation

**Figure 7:**
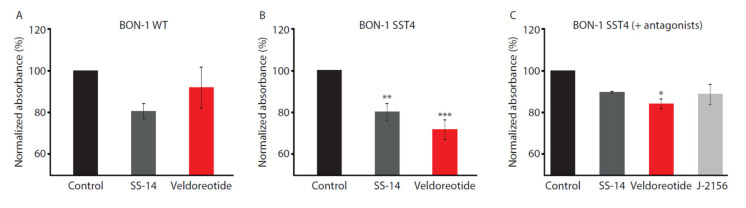
Cell proliferation in (**A**) wild-type BON-1 cells or BON-1 cells expressing SST4 receptor following treatment with (**B**) 10 µM SS-14 or (**C**) 10 µM veldoreotide for 24 h at 37 °C. In C, SST4 receptor agonist J-2156 was compared with SS-14 and veldoreotide. Antagonists to the SST2 receptor (BIM23454) and SST5 receptor (BIM23867) were used. Data shown were generated from three to four independent experiments performed in duplicate (mean ± SEM). Absorbance data for cells treated with agonists were normalized to vehicle (control). * *p* < 0.05 vs. control; ** *p* < 0.01 vs. control; *** *p* < 0.001. SEM = standard error of the mean.

The effect of veldoreotide on cell proliferation was examined in the wild-type BON-1 cells, which express the endogenous SST2 and SST5 receptors, and in the BON-1 cells stably expressing the SST4 receptor. In the wild-type BON-1 cells, SS-14 and veldoreotide inhibited proliferation to a similar extent (Figure 7). However, in the BON-1 cells expressing the exogenous SST4 receptor, veldoreotide inhibited cell proliferation to a greater degree than SS-14 (71.2% vs. 79.7%, respectively). The addition of the SST2 and SST5 receptor antagonists BIM23454 and BIM23867, respectively, to SS-14, veldoreotide, or the SST4 receptor agonist J-2156 demonstrated that the inhibitory activity of veldoreotide on cell proliferation was also mediated via the SST4 receptor. Similarly, the SS-14 or J-2156 effects on cell proliferation in the presence of the SST2 and SST5 receptor antagonists were comparable with those observed for veldoreotide.

## 4. Discussion

The findings of this study indicate that veldoreotide is an SSA with full agonist activity at the SST4 receptor along with the SST2 and SST5 receptors as assessed by a fluorescence-based membrane potential assay in a HEK293 human embryonic kidney cell line and a BON-1 human pancreatic neuroendocrine tumor cell line. As the GIRK channels are not expressed endogenously in the HEK293 cells [24], these cells were stably cotransfected with the GIRK2-encoding plasmid. The efficacy (E_max_ values) of veldoreotide in activating the SST4 receptor was comparable with that of the endogenous SS-14, and for the SST2 and SST5 receptors, it was comparable with that of octreotide and pasireotide. The antisecretory and antiproliferative activities of veldoreotide mediated through the SST4 receptor were demonstrated by a comparison with the activities of SS-14 in the BON-1 cells overexpressing the SST4 receptor. In addition, veldoreotide could still inhibit cell proliferation in the BON-1 cells stably expressing the SST4 receptor when the endogenous SST2 and SST5 receptors were blocked with their respective antagonists, further corroborating the agonist activity of veldoreotide at the SST4 receptor.

The implications of veldoreotide activity at the SST4 receptor are multifold, albeit not entirely clear, given, in part, the lack of antibodies specific to the human SST4 receptor [2]. However, the clinical utility of an SSA that binds to the SST4 receptor is promising in light of the widespread expression of the SST4 receptor from the brain to the immune system [9,10,11,12,13,14,15]. Upregulation of SST4 receptors in patients with chronic pulmonary inflammation suggests that the SST4 receptor agonists may have therapeutic potential in inflammatory lung conditions [11]. Further, veldoreotide is unique in that it is an SSA with activity at the SST4 receptor along with agonist activity at the SST2 and SST5 receptors, which may point towards an efficacy for conditions unresponsive to currently available SSAs. A multireceptor-targeting SSA such as veldoreotide can potentially provide higher antitumor activity compared with SSAs with agonist activity for a single SST receptor [7]. Indeed, veldoreotide inhibited growth hormone secretion in a greater percentage of adenomas compared with octreotide, including adenomas nonresponsive to octreotide treatment. Based on the differentiated activation pattern of SST-receptor subtypes, veldoreotide may have potential applications in endocrine and nonendocrine conditions that are amenable to SST receptor activation.

Interestingly, animal studies have demonstrated the importance of the SST4 receptor in pain modulation [16,17]. Given the preliminary findings regarding the comparable antiproliferative activity of veldoreotide and the SST4 receptor agonist J-2156 [29] in BON-1 pancreatic neuroendocrine cells transfected with the SST4 receptor in this study, it is possible that veldoreotide and J-2156 may have similar physiological activities in humans. J-2156 has been shown to alleviate lower back pain and breast cancer-induced bone pain in preclinical animal studies [18,30]; therefore, the potential for veldoreotide as a pain modulating agent holds promise. Additional studies are warranted to test this hypothesis.

Continued examination of veldoreotide will further elucidate the role of the SST4 receptor in physiological processes and in various medical conditions. The current analyses are limited to the characterization of veldoreotide at the SST4 receptor using stably transfected cell lines, which are an acceptable preclinical model for characterizing binding and specific activities of a novel agent. Knock-out animal models could test the hypothesis that veldoreotide may have a role in modulating pain.

## 5. Conclusions

To the best of our knowledge, this is the first preclinical study to demonstrate that the novel SSA veldoreotide has agonist activity at the SST4 receptor. Veldoreotide has potential applications for conditions in which the SST4 receptor plays a role, as well as in patients who are currently unresponsive to other SSAs.

## Figures and Tables

**Table 1:** Agonist-selective G-protein signaling in HEK293 cells.

	GIRK2-SST2	GIRK2-SST4	GIRK2-SST5
SS-14 ^a^			
E_max_, %	100 ± 3.2	100 ± 6.8	100 ± 4.4
EC_50_, nM	2.0 ± 0.9	5.5 ± 2.5	6.6 ± 1.5
Octreotide			
E_max_, %	99.0 ± 2.4	27.4 ± 4.7	92.0 ± 5.7
EC_50_, nM	4.6 ± 0.6	32.8 ± 39.6	54.4 ± 6.6
Pasireotide			
E_max_, %	102.2 ± 2.8	52.0 ± 4.9	98.6 ± 3.9
EC_50_, nM	83.7 ± 8.1	222.9 ± 115.8	16.5 ± 2.5
Veldoreotide			
E_max_, %	98.0 ± 2.9	96.9 ± 2.7	90.4 ± 5.2
EC_50_, nM	37.6 ± 4.5	31.3 ± 14.4	10.5 ± 3.4
J-2156			
E_max_, %	–	100.7 ± 5.1	–
EC_50_, nM		1.6 ± 0.5	

Mean ± standard error of the mean calculated using duplicate determinations from three to four independent experiments. ^a^ SS-14 is an endogenous SST ligand. EC_50_ = half-maximal effective concentration; E_max_ = maximum effect; GIRK = G-protein-coupled inwardly rectifying potassium channels; HEK = human embryonic kidney; SST = somatostatin.

**Table 2:** Agonist-selective G-protein signaling in BON-1 cells.

	Wild Type	SST2	SST4	SST5
SS-14 ^a^				
E_max_, %	100 ± 5.5	100 ± 7.4	100 ± 4.0	100 ± 1.6
EC_50_, nM	80.9 ± 36.8	1.6 ± 0.6	16.5 ± 4.6	2.2 ± 0.5
Veldoreotide				
E_max_, %	31.4 ± 3.0	83.1 ± 0.9	96.5 ± 3.5	93.3 ± 2.5
EC_50_, nM	41.7 ± 28.2	19.0 ± 4.2	28.3 ± 14.2	16.6 ± 2.7
J-2156				
E_max_, %	–	–	97.5 ± 2.1	–
EC_50_, nM			1.0 ± 0.3	

Mean ± standard error of the mean calculated using duplicate determinations from four independent experiments. ^a^ SS-14 is an endogenous SST ligand. EC_50_ = half-maximal effective concentration; E_max_ = maximum effect; SST = somatostatin.

## Data Availability

All relevant data are included in this article and data sharing is not applicable.

## Supplementary Materials

The following are available online at https://www.mdpi.com/article/10.3390/life11101075/s1, Figure S1: Stable expression of SST receptors with GIRK2 expression in HEK293 cells, Figure S2: SS14-induced fluorescent traces from HEK293 cells expressing SST receptors and GIRK2.

## Published Materials

The results/data/figures in this manuscript have not been published previously, nor are they under consideration for publication elsewhere. However, part of the data were presented at ENDO: Endocrine Society 2020; March 28–31; San Francisco, CA, USA.
