# Supplementary material for: Pharmacological Characterization of Veldoreotide as a Somatostatin Receptor 4 Agonist
Source: Life (Basel). 2021 Oct 12;11(10):1075. doi: 10.3390/life11101075 (PMC8541358; doi:10.3390/life11101075)
Supplement: Supplementary file 1 [file life-11-01075-s001.zip › life-1372048-supplementary.pdf]

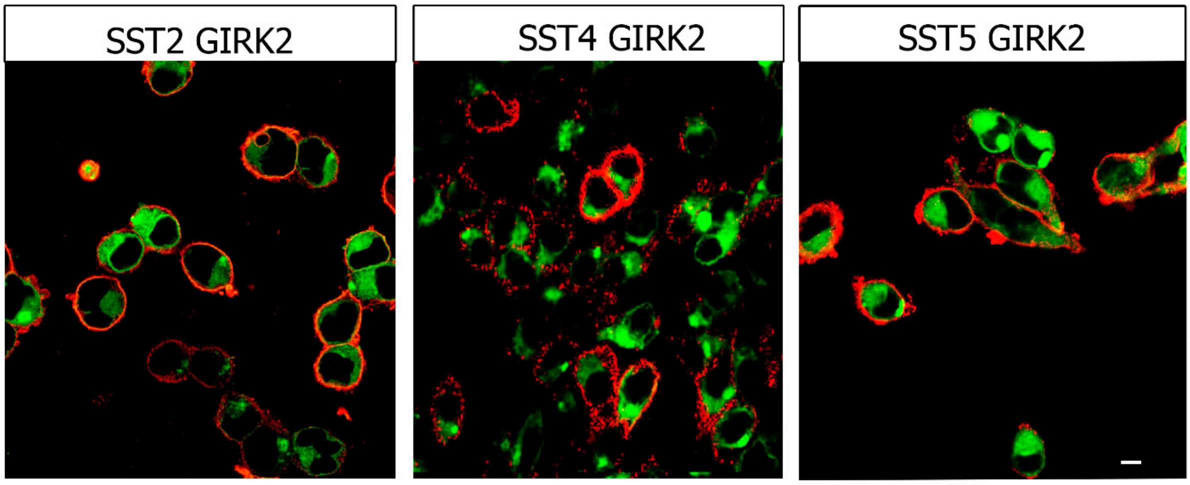


**Figure S1.** Stable expression of SST receptors with GIRK2 expression in HEK293 cells. Red staining (Cy3) confirms SST receptor expression in cells and green color represents GIRK2 expression. 40× magnification; bar = 20 µm. GIRK = G protein-coupled inwardly rectifying potassium; SST = somatostatin.

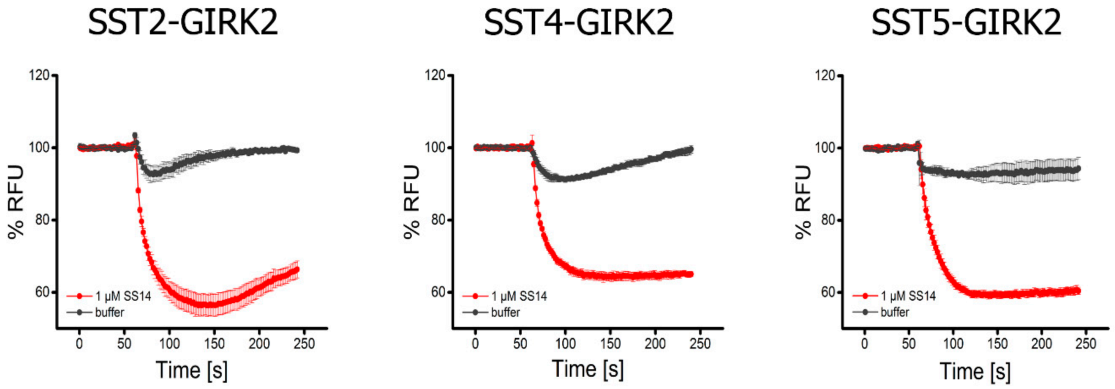


**Figure S2.** SS14-induced fluorescent traces from HEK293 cells expressing SST receptors and GIRK2. GIRK = G protein-coupled inwardly rectifying potassium; RFU = relative fluorescence units; SST = somatostatin.
